# Cross‐Linked Volume‐Stable Collagen Matrix Versus Connective Tissue Graft for Soft Tissue Augmentation at Implant Site. A Non‐Inferiority, Multicenter Randomized Clinical Trial

**DOI:** 10.1111/clr.70050

**Published:** 2025-09-22

**Authors:** Francesco Cairo, Cosimo Rupe, Raffaele Cavalcanti, Luca Landi, Antonio Rupe, Nicola Marco Sforza, Walter Castelluzzo, Maria Di Martino, Luigi Barbato

**Affiliations:** ^1^ EFP Master Program in Periodontology and Implant Dentistry and Research Unit in Periodontology and Periodontal Medicine, Department of Clinical and Experimental Medicine University of Florence Florence Italy; ^2^ Private Practice in Bari Bari Italy; ^3^ Private Practice in Roma and Verona Verona Italy; ^4^ Private Practice in Benevento Benevento Italy; ^5^ Private Practice in Bologna Bologna Italy

**Keywords:** connective tissue graft, dental implants, keratinized tissue, randomized clinical trial, soft tissue thickness, volume stable collagen matrix

## Abstract

**Aims:**

To compare the efficacy of cross‐linked volume‐stable collagen matrix (VCMX) versus connective tissue graft (CTG) to increase buccal peri‐implant mucosal thickness (MT) around dental implants.

**Methods:**

The present is a parallel, randomized multi‐center clinical trial, according to the CONSORT statement. Clinical centers were four Italian periodontal settings. All patients received a soft tissue augmentation procedure, by means of CTG or VCMX. The primary outcome variable was peri‐implant mucosal thickness (MT) difference at 12 months follow‐up. The statistical unit was the patient. An analysis of covariance was performed for this outcome variable. Secondary outcomes were patient‐reported outcome measures (PROMs), complications, variability among operators, and changes in keratinized mucosa width (KMW).

**Results:**

A total of 98 patients completed the study, 49 in each group. MT increase was 1.0 ± 0.75 in the CTG group and 0.66 ± 0.58 mm in the VCMX group. CTG showed superior results to VCMX for MT gain (0.37 mm, 95% CI: 0.13–0.61, *p* = 0.002). In cases of Baseline MT ≥ 2 mm, CTG and VCMX yielded comparable results. VCMX was associated with shorter chair time (diff: 10.0 min; 95% CI: 5.02 to 14.98; *p* < 0.0001). Patients in the VCMX group experienced fewer days of discomfort (0.46 days, 95% CI: 0.06–0.99, *p* = 0.05), while no differences were found for final aesthetic and general satisfaction (CTG: 99.28 ± 2.28, VCMX: 98.48 ± 3.92, *p* = 0.22).

**Conclusions:**

Both techniques improved MT, and CTG yielded better outcomes. VCMX was associated with shorter chair time and less postoperative discomfort, but both procedures achieved excellent final patient satisfaction.

## 1 Introduction

Although a common paradigm suggests that the quality of peri‐implant mucosa is not able to influence implant survival by itself (Wennström et al. 1994) (Bengazi et al. 1996), a careful management of soft tissue around implants may be considered a key factor to obtain aesthetic outcomes and to support an easier long‐term maintenance (Cairo et al. 2008).

An increasing body of evidence suggests that soft tissue augmentation procedures, with the aim to improve either peri‐implant mucosal thickness (MT) or the amount of keratinized mucosa width (KMW) (Avila‐Ortiz et al. 2020) (Thoma et al. 2018) (Tavelli et al. 2021) (Stefanini et al. 2023), have a beneficial impact on peri‐implant health and marginal bone level (MBL) stability and may favor the primary prevention of peri‐implant diseases (Carra et al. 2023).

Soft tissue augmentation procedures might have a valuable effect on the final aesthetic results of implant‐supported restorations (Bienz et al. 2022) and may prevent soft tissue recession at implant sites (Cairo, Barootchi, et al. 2020).

Several periodontal plastic surgery techniques may be used to augment soft tissue thickness (Cairo et al. 2008), with the connective tissue graft (CTG) being the gold standard (Cairo et al. 2017) (Valles et al. 2022).

However, this technique has some disadvantages, such as the associated morbidity. To overcome these limitations, xenogeneic collagen matrix (XCM) has been proposed as an alternative material for soft tissue augmentation.

Preliminary clinical studies showed that XCM may be an effective alternative to CTG without the morbidity of soft tissue graft harvest in the treatment of gingival recession (McGuire and Scheyer 2010) (Thoma et al. 2012). A randomized clinical trial (RCT) also compared also XCM to CTG to increase soft tissue volume around dental implants, showing that XCM is associated with a significant increase in MT compared with baseline (Cairo et al. 2017).

Recently, a modification of XCM was introduced to obtain a cross‐linked volume‐stable collagen matrix (VCMX), and a pilot study suggested that this matrix may provide similar benefits to CTG in terms of MT (Thoma et al. 2016).

Consequently, the interest in clinical research towards soft tissue substitutes has increased, and some clinical trials highlighted their potential (Hämmerle et al. 2023) (Clem et al. 2024) (Surdiacourt et al. 2025). Nevertheless, it remains inconclusive whether soft tissue augmentation using a VCMX is noninferior to CTG in terms of MT increase at implant sites.

The aim of this multicenter RCT was to assess and compare the efficacy of VCMX versus CTG around dental implants in terms of MT augmentation, patient‐reported outcome measures (PROMs), complications, and variability among operators.

## 2 Materials and Methods

### 2.1 Trial Design

The present study is a parallel, randomized multi‐center clinical trial with a 1:1 allocation ratio, conducted according to the CONSORT statement (http://www.consort‐statement.org/).

The study was performed according to the ISO Standard 14,155:2011 in clinical investigations using medical devices in human patients (appendices VIII and X of the Medical Device Directive 93/42/EEC and with the Declaration of Helsinki 2013). The study was authorized by the Ethical board CEAVC (Comitato Etico Area Vasta Centro, Toscana, Italia): ethical approval number: n 16434_spe, and registered on clinicaltrials.gov (NCT05458271). The participants had to sign the informed consent.

Two different procedures to increase buccal MT at the implant site were compared: volume stable xenogeneic collagen matrix (VCMX) (test group) and connective tissue graft (CTG) (control group).

### 2.2 Participants

#### 2.2.1 Inclusion Criteria

- Age ≥ 21 years.
- Self‐reported smoking ≤ 20 cigarettes/day.
- No probing depths ≥ 5 mm at the remaining dentition.
- Full‐mouth plaque score (FMPS) and full‐mouth bleeding score (FMBS) ≤ 15%.
- Need of peri‐implant mucosal thickness (MT) augmentation for aesthetic purposes and/or functional reasons at upper or lower jaw.
- KMW ≥ 2 mm at the site of MT augmentation.
- No previous soft tissue augmentation procedure at experimental site.

Considering that data from literature suggested no significant difference between one and two stage implants in terms of gingival margin and bone level positions after healing of surgery with the apical positioned flap (Small and Tarnow 2000; Oates et al. 2002), both one and two stage implants were considered in the present study.

#### 2.2.2 Exclusion Criteria

- General contraindications for dental and/or surgical treatments.
- Concurrent or previous immunosuppressant, bisphosphonate, or high‐dose corticosteroid therapy.
- Inflammatory and autoimmune diseases of the oral cavity.
- Radiotherapy of head and neck area.
- Any systemic diseases or conditions affecting connective tissue or bone metabolism.
- Untreated periodontitis.
- Dental implants with a pre‐existing healing abutment.
- Type 1 implant placement (Hämmerle et al. 2004).

### 2.3 Interventions

#### 2.3.1 Surgical Procedure

Following the local anesthesia, a flap was raised, and the implant was placed or uncovered, applying a healing abutment. Care was taken to preserve the pre‐existing KMW amount, making sure that the flap, in its final position, retained at least 2 mm of KMW buccal to the implant. A mesio‐distal and apical partial thickness dissection was performed to allow the passive displacement of the flap. The randomization sealed and opaque envelope was opened, and the patient was allocated to the test or control group. In the test group, VCMX (Fibrogide, Geistlich Pharma AG, Wolhusen, Switzerland) with an initial dimension of 15 × 20 × 6 mm was gently shaped and secured under the flap, as deemed appropriate by the surgeon. VCMX thickness was never reduced, keeping a standard 6 mm dimension. In the control group, a standard 1‐mm thick CTG (harvested at the palatal side) was adjusted according to the defect site dimensions and sutured at the buccal supra‐periosteal tissue. The flap was then sutured, applying the maximum care to completely cover the graft. The soft tissue augmentation procedure was not extended at neighboring teeth. In the case of soft tissue augmentation contextual to implant placement, a transmucosal approach was chosen in each of the treated cases.

#### 2.3.2 Post‐Surgical Instruction, Controls and Professional Tooth Cleaning

Post‐operative pain/oedema was controlled with anti‐inflammatory drugs (ibuprofen 600 mg). Patients were instructed to assume two tablets during the first day of healing. Subsequent doses were taken only when necessary. Smokers were reminded to limit (and possibly avoid) smoking.

All patients were instructed to discontinue tooth brushing and avoid any trauma at the surgical site. Chlorhexidine digluconate was prescribed 3×/day for the first 2 weeks.

**FIGURE 1:**
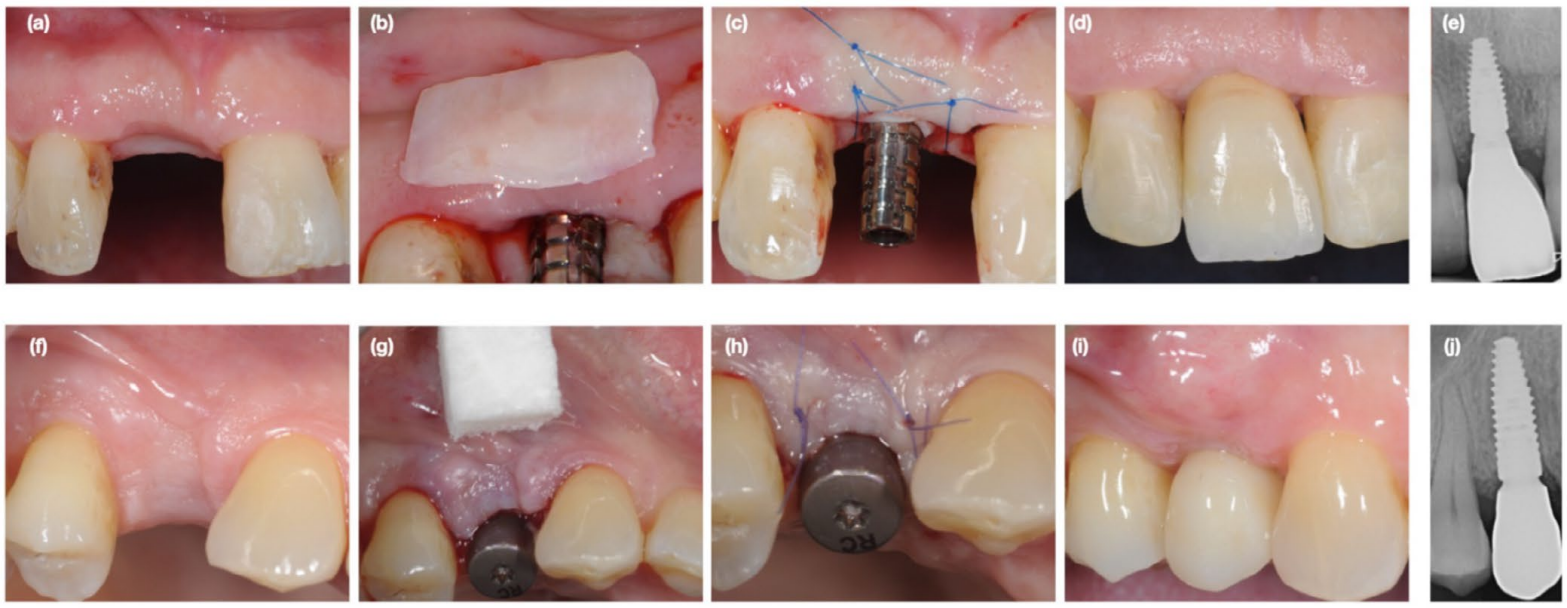
(a–e) Clinical example of a case allocated to CTG group (a) Baseline of experimental procedure; (b) clinical case allocated to CTG; (c) flap suture over CTG; (d) final soft tissue healing at the last follow‐up; (e) final X‐ray. (f, g) Clinical example of a case allocated to VCMX group. (f) Baseline of experimental procedure. (g) Clinical case allocated to VCMX. (h) Flap suture over VCMX. (i) Final soft tissue healing at the last follow‐up. (j) Final X‐ray. CTG, connective tissue graft; VCMX, volume stable collagen matrix.

The sutures were removed after 14 ± 1 days. Patients were recalled for controls at Weeks 1, 2, 4, and at months 3, 6, and 12. One month after experimental procedures, prosthetic treatments started by applying a temporary crown. The final impression for the application of the definitive abutment and crown was scheduled 3 months after the experimental treatment, but the clinicians were allowed to customize the prosthetic procedure (i.e., extend the duration of temporary prosthetic treatment, apply more than one provisional restoration) in order to reach satisfactory soft tissue conditions (e.g., scalloped gingival margin, soft tissue contour). Clinical examples of treated cases are provided in Figure 1.

#### 2.3.3 Operators Training

The clinical centers were four Italian periodontal clinical centers, and the operators were expert surgeons with more than 10 years of experience, all active members of the Italian Society of Periodontology (SIdP). The surgeons attended a training and calibration session before the start of the trial.

### 2.4 Outcomes

#### 2.4.1 Baseline Variables

Baseline data collection included demographic variables: age, sex, medications, smoking habits, periodontal diagnosis, and previous bone augmentation at the experimental site.

#### 2.4.2 Clinical and Radiographic Variables

The following measurements were taken for each treated implant:

- Primary outcome
  ○ MT: buccal peri‐implant mucosal thickness, measured 1 mm coronal to the MGJ using an injection needle, perpendicular to the tissue surface, with a silicon stop over the gingival surface. The silicon disk stop was placed in tight contact with the soft tissue surface and fixed with a drop of cyanocrylic adhesive. After needle removal, the distance between the needle tip and the silicon stop was measured using a digital caliper with 0.01 mm of accuracy (Cairo et al. 2016). The measurement was repeated at the 1‐, 3‐, 6‐, and 12‐month follow‐up.
- Secondary outcomes
  ○ KMW: buccal keratinized mucosa, measured as the distance between muco‐gingival junction (MGJ) and the most coronal point of the ridge, using a periodontal probe (PCP UNC 15, Hu‐Friedy). The measurement was repeated 7 and 14 days after the surgical procedure and at the 1, 3, 6, and 12‐month follow‐up. After the placement of the prosthetic restoration, KMW was measured as the distance between MGJ and peri‐implant mucosal margin.
  ○ MBL: distance in mm between bone levels at the mesial and distal sites of the experimental implant using the first thread as a reference point. This measurement was obtained after the application of the healing abutment using an intra‐oral X‐ray obtained with the parallel technique. The measurements were rated positive when the bone crest (BC) was placed coronal to the first implant thread and negative when BC was below the reference point, and were performed at baseline, 6‐ and 12‐month follow‐up.
  ○ Soft tissue margin recession (REC): Presence or absence of soft tissue margin recession at 6 points for implant, defined as the distance between implant shoulder and gingival margin at temporary or final crown. REC was defined to be present when the abutment margin location was in supramucosal position. It was assumed that when the abutment–crown junction was not visible, there was no soft tissue recession.
  ○ Probing depth (PD): at 6 points for implant.
  ○ Bleeding on probing (BoP): as yes/no at 6 points/implant

For patients allocated in the control group (CTG), the type of harvesting procedure was described, and the chair time of the surgical procedure was measured from the end of local anesthesia until the completion of the last suture.

Data on possible soft tissue complications (necrosis, edema, bleeding) were collected.

#### 2.4.3 Patient‐Reported Outcome Measures (PROMs)

Immediately after the end of the surgical procedure, patients were asked by interview about the hardship of the procedure and the pain perceived during the procedure. All patients received a questionnaire that was asked to complete throughout the post‐operative period, in order to assess the extent of their pain or discomfort using a visual analogue scale (VAS from 0 to 100), to record the duration of the pain or discomfort (number of days), number of tablets of painkillers taken (total number), side effects, interference with daily activities (Yes/No).

At the time of suture removal, these variables were recorded. Possible complications were checked and reported at every follow‐up. At the final visit, overall/aesthetic patient satisfaction was assessed using a VAS scale.

#### 2.4.4 Evaluation of Soft Tissue Volume

**FIGURE 2:**
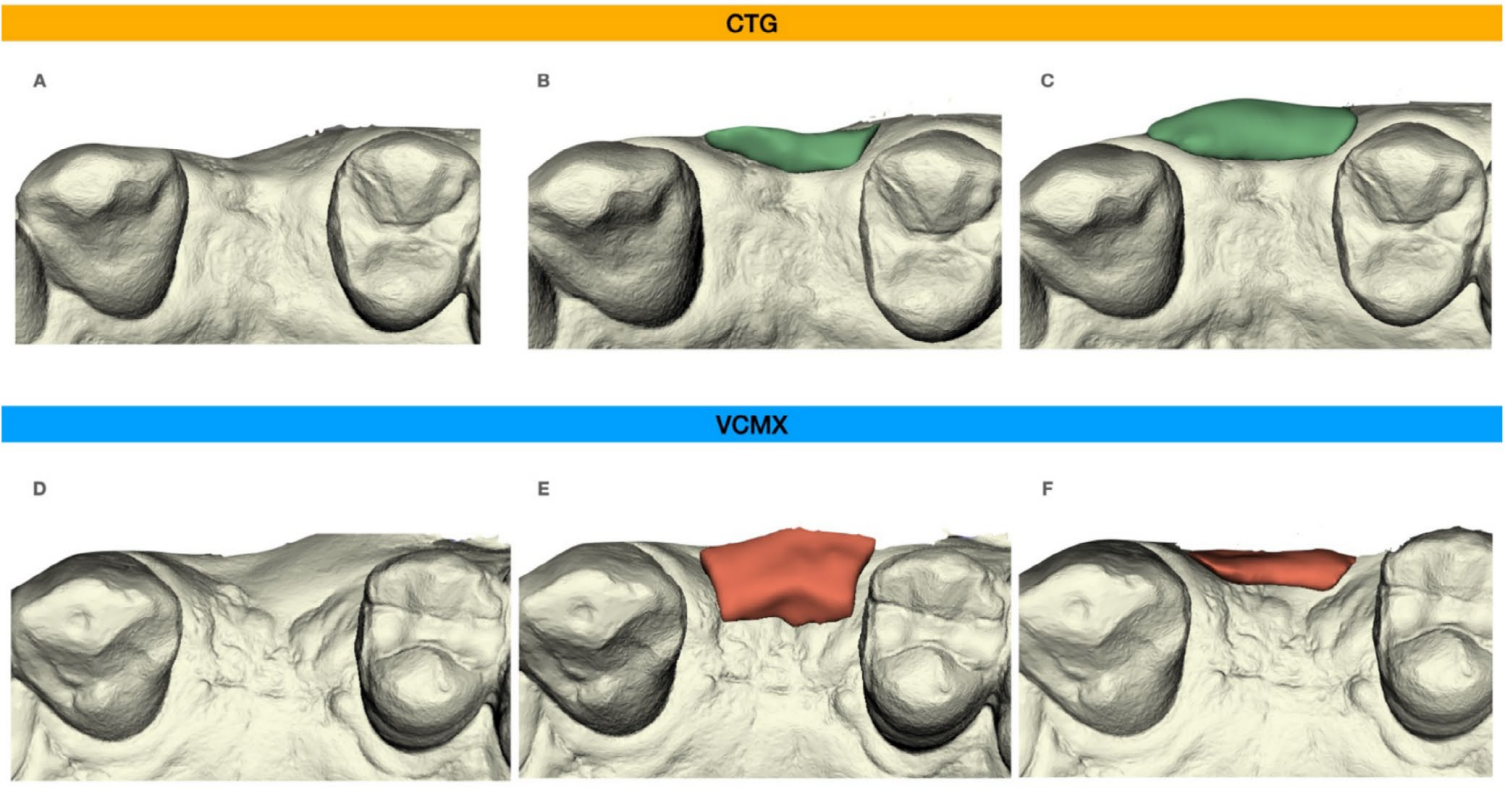
(a) Baseline of experimental procedure. (b) Volumetric and profilometric changes in the area of interest between T0 (white) and T1 (green) at the 6 months follow‐up. (c) Volumetric and profilometric changes in the area of interest between T0 (white) and T1 (green) at the 12 months follow‐up. (d) Baseline of experimental procedure. (e) Volumetric and profilometric changes in the area of interest between T0 (white) and T1 (orange) at the 6 months follow‐up. (f) Volumetric and profilometric changes in the area of interest between T0 (white) and T1 (orange) at the 12 months follow‐up. CTG, connective tissue graft; VCMX, volume stable collagen matrix.

To evaluate the dimensional changes at the experimental sites, intra‐oral scans (Trios, 3shape, Copenhagen, Denmark) were taken at the following time points: baseline, 3, 6, and 12 months. The images representing the different treatment time points of examination were superimposed and matched using the cusps of the neighboring and contra‐lateral teeth as reference points. Subsequently, the area of interest at each defect site was measured, and the volume difference between the time points was calculated. To allow a direct comparison of the different sites, the calculated variable represented the volume difference per measured area. Volume difference was calculated between Day 0 and the final examination (12 months). All the 3D analyses were performed by an author (C. R.), who was not involved in any surgical or prosthetic procedure, by means of the 3D Slicer image computing platform (https://www.slicer.org/). Figure 2 illustrates an example of volumetric and profilometric changes in soft tissue volume, illustrating the behavior of the two techniques in the majority of the cases.

### 2.5 Sample Size

Primary outcome was MT changes after a 12 months follow‐up. Considering a non‐inferiority study design, the sample dimension was calculated using *α* = 0.05 and the power of the sample (1−*β*) = 80%. For the variability (*σ* difference SD = 0.63 mm), a standard deviation obtained in a previous paper (Wiesner et al. 2010) was considered. The minimum clinically significant value (*δ*) considered was 0.4 mm. Sample size calculation was performed by means of the online software https://www.trialdesign.org/.

Based on these data, the needed number of patients to be enrolled to conduct this study has been estimated to be 90 patients. To compensate for potential dropouts, the number of patients was increased by 10%, leading to a final sample of 100 patients to be included in the trial.

### 2.6 Randomization and Allocation Concealment

Each experimental subject was randomly assigned to one of the two treatment regimens. A blocked randomization was used. Treatment assignment was noted in the registration and treatment assignment form kept by the study registrar (L.B.), who was not involved in any of the surgical procedures. Allocation concealment was performed by opaque sealed envelopes, which were sequentially numbered. The allocation sequence was generated by means of a computer‐generated random list. The opaque envelopes were opened after flap elevation, and treatment assignment was then communicated to the operator.

### 2.7 Blinding

For each experimental center, an examiner, masked with respect to treatment allocation, not involved in the clinical procedures, performed the measurements. The investigators were trained for MT evaluation, participating in a 2‐day calibration meeting. Examiners had to achieve an intra‐examiner reproducibility > 79% within 0.1 mm for MT in 10 non‐study‐related subjects.

### 2.8 Statistical Analysis

Descriptive statistics were performed using mean ± standard deviation for quantitative variables and frequency and percentage for qualitative variables. The statistical unit was the patient. Differences between groups for quantitative variables were assessed by the Student's *t*‐test in the case of normally distributed variables, whereas the Kruskal–Wallis test was used in the case of a non‐normal distribution. Differences in qualitative variables were assessed by the *χ* ^2^ test or Fisher's exact test, as appropriate. The primary outcome variable was peri‐implant mucosal thickness (MT) difference, considered as the difference between MT at baseline and MT at the 12‐month follow‐up. An analysis of covariance was performed for this outcome variable using treatment, experimental center, and timing of soft tissue augmentation (i.e., at implant placement or implant uncovering) as explicative variables and MT T0 as a covariate. The interaction terms were added to the model if significant. The analysis of covariance was also performed for the difference in apico‐coronal KMW comparing baseline and 12 months' follow‐up. ANOVA test was performed for chair time, number of post‐operative painkillers, number of days with discomfort, PD, number of BOP sites, VAS regarding final aesthetic and overall satisfaction. This analysis was defined a priori. Drop‐out patients were excluded from the statistical analysis.

## 3 Results

### 3.1 Experimental Population, Patients and Defects Characteristics at Baseline

**FIGURE 3:**
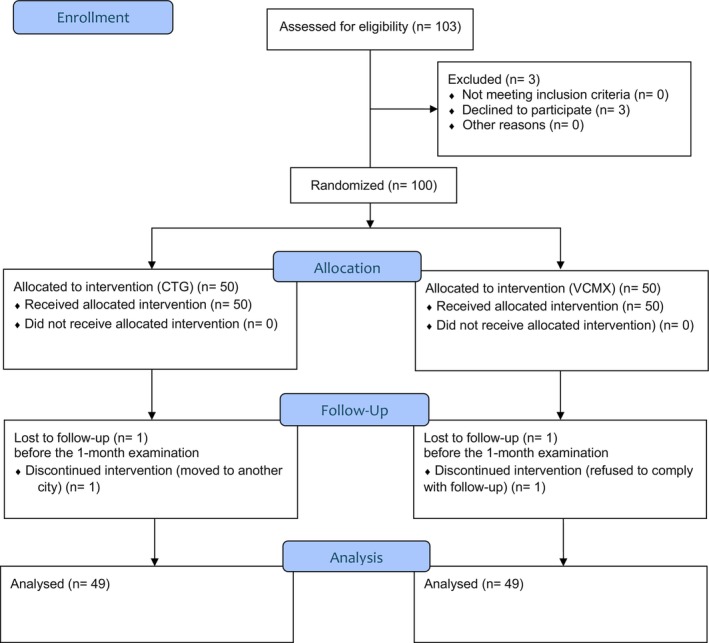
CONSORT flow‐diagram of the study.

A total of 100 patients participated in the study, 50 in each group, while 98 patients (49 in each group) completed the study (Figure 3). Demographics and patients' characteristics are reported in Table 1. Patients were consecutively recruited in the clinical centers from July 2022 to October 2023.

**TABLE 1:** Demographic characteristics of the included patients.

Variable	CTG	VCMX
	*N* = 49	*N* = 49
Sex		
Female	27 (55.1%)	26 (53.1%)
Male	22 (44.9%)	23 (46.9%)
Age (years) − Mean (SD)	56.7 (10.3)	59.3 (11.5)
Systemic diseases (yes)	16 (32.6%)	15 (30.6%)
Periodontitis (yes)	36 (73.5%)	37 (75.5%)
Stage of periodontitis		
Stage 1	3 (6.1%)	2 (4.1%)
Stage 2	7 (14.3%)	9 (18.4%)
Stage 3	17 (34.7%)	20 (40.8%)
Stage 4	9 (18.4%)	6 (12.2%)
Grade of periodontitis		
Grade A	2 (4.1%)	0 (−)
Grade B	18 (36.7%)	22 (44.9%)
Grade C	16 (32.6%)	15 (30.6%)
Extent of periodontitis		
Generalized	31 (63.3%)	32 (65.3%)
Smoking (yes)	13 (26.5%)	10 (20.4%)
Smoking (≤ 10 cig)	11 (22.4%)	7 (14.3%)
Smoking (> 10 cig)	2 (4.1%)	3 (6.1%)
Reason for tooth loss		
Periodontitis	17 (34.7%)	13 (26.5%)
Fracture	10 (20.4%)	16 (32.6%)
Caries	11 (22.4%)	9 (18.4%)
Endodontic failure	6 (12.2%)	4 (8.2%)
Other	5 (10.2%)	7 (14.3%)
Implant brand and characteristics		
Straumann bone level	31 (63.2%)	26 (53.1%)
Straumann tissue level	14 (28.6%)	20 (40.8%)
Biomet 3i	4 (8.2%)	3 (6.1%)
Experimental site		
Maxillary central incisor	9 (18.4%)	1 (2%)
Maxillary lateral incisor	2 (4.1%)	1 (2%)
Maxillary canine	1 (2%)	0 (−)
Maxillary premolar	20 (40.8%)	20 (40.8%)
Maxillary molar	4 (8.2%)	2 (4.1%)
Mandibular incisor	3 (6.1%)	4 (8.2%)
Mandibular premolar	2 (4.1%)	6 (12.2%)
Mandibular molar	8 (16.3%)	15 (30.6%)
Previous bone augmentation		
No	25 (51.1%)	34 (69.4%)
ARP	11 (22.4%)	8 (16.3%)
GBR	13 (26.5%)	7 (14.3%)
Timing of soft tissue augmentation procedure		
Implant placement	18 (36.7%)	21 (42.9%)
Implant uncovering (second surgery)	31 (63.3%)	28 (57.1%)
Type of implant‐supported restoration		
Screw‐retained	49 (100%)	49 (100%)
Single crown	32 (65.3%)	31 (63.3%)
Fixed partial denture	17 (34.7%)	18 (36.7%)
MBL (mm) − Mean (SD)	1.1 (0.9)	0.94 (0.89)
KMW baseline (mm) − Mean (SD)	3.61 (1.2)	3.44 (1.1)
MT (mm) − Mean (SD)	2.2 (1.1)	2.2 (1.2)

Abbreviations: ARP, alveolar ridge preservation; CTG, connective tissue graft; GBR, guided bone regeneration; KMW, keratinized mucosa width; MBL, marginal bone loss; MT, peri‐implant mucosal thickness; SD, standard deviation; VCMX, volume stable collagen matrix.

In the CTG group, 27 out of 49 patients were females (55.1%), and the mean age was 56.7 ± 10.3 years. Thirteen patients were smokers (26.5%). The mean baseline MT was 2.2 ± 1.1 mm.

In VCMX group, 26 out of 49 were females (53.1%), and the mean age was 59.3 ± 11.5 years. Ten patients were smokers (20.4%). The mean baseline MT was 2.2 ± 1.2 mm. There was no statistically significant difference at baseline between the two groups.

### 3.2 Outcomes and Estimation

#### 3.2.1 Clinical and Radiographic Outcomes

Descriptive statistics of the following variables: KMW, MT, MBL, PPD, REC, BoP for the test and control groups are presented in Table 2.

**TABLE 2:** Descriptive statistics at baseline, 3‐, 6‐, and 12‐months regarding KMW and MT.

Variable	CTG	VCMX	CTG	VCMX	CTG	VCMX	CTG	VCMX	*p*
	T0		3 months		6 months		12 months		
KMW	3.63 (1.3)	3.42 (1.1)	4.2 (1.5)	3.85 (1.2)	4.19 (1.3)	3.72 (1.3)	4.55 (1.4)	3.57 (1.3)	**< 0.0001**
MT	2.20 (1.1)	2.20 (1.2)	2.93 (0.9)	3.34 (1.0)	3.08 (1.0)	3.01 (0.89)	3.27 (1.0)	2.88 (0.9)	**0.002**
MBL	1.1 (1.0)	0.94 (0.87)	—	—	0.69 (0.91)	0.56 (0.89)	0.47 (0.80)	0.43 (0.98)	0.88
PPD	—	—	—	—	2.79 (0.88)	2.64 (0.85)	2.98 (0.84)	2.66 (0.83)	0.08
REC (percentage of sites)	—	—	—	—	0%	6.1%	0%	8.2%	0.12
BoP (percentage of sites)	—	—	—	—	4.76%	6.8%	6.12%	6.8%	0.55

*Note:* Bold *p*‐values indicate statistically significant difference between groups at the 12‐month follow‐up.

Abbreviations: BoP, bleeding on probing; CTG, connective tissue graft; KMW, keratinized mucosa width; MT, peri‐implant mucosal thickness; PPD, pocket probing depth; REC, recession of the soft tissue margin; VCMX, volume stable collagen matrix.

No significant complication was reported. No peri‐implantitis was diagnosed, while 9 cases of peri‐implant mucositis were diagnosed (9.2%). No significant difference was also reported in terms of MBL at the last follow‐up (difference 0.04 mm; 95% CI: −0.4 to 0.3; *p* = 0.8).

**FIGURE 4:**
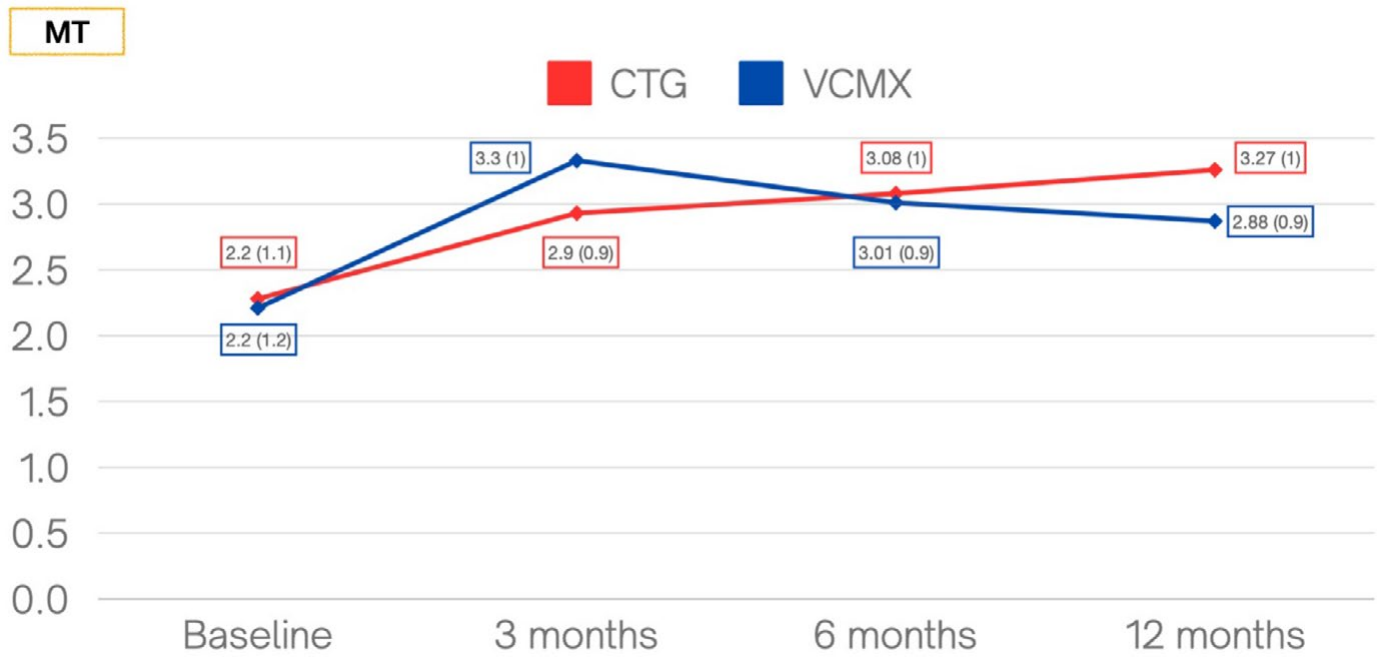
Graphic description of measurements for MT at each time interval. All values are expressed in mm. CTG, connective tissue graft; MT, peri‐implant mucosal thickness; VCMX, volume stable collagen matrix.

MT changes at each interval are presented in Figure 4. At the 12‐month follow‐up visit, both procedures resulted in a significant increase in MT compared with baseline (*p* < 0.0001). Final MT was 3.27 ± 1.0 in the CTG group and 2.88 ± 0.9 mm in the VCMX group.

MT increase was 1.0 ± 0.75 in the CTG group and 0.66 ± 0.58 mm in the VCMX group.

The mean volumetric changes in ridge contour between baseline and 12 months amounted to 47.63 mm^3^ ± 29.8 in the CTG group and to 27.28 mm^3^ ± 19.63 in the VCMX group (*p* = 0.03).

After adjusting for initial MT, CTG showed superior results compared to VCMX in terms of MT changes (0.37 mm, 95% CI: 0.13–0.61, *p* = 0.002), and the variable “center” significantly impacted the final outcomes. The increase in MT was higher for initially thinner soft tissues. On the other hand, the timing of soft tissue augmentation did not significantly affect final MT gain (*p* = 0.515) (Table 3).

**TABLE 3:** Results of the Multivariate analysis for change in MT between baseline and 12 months follow‐up.

Variables	Estimate	Standard error	95% CI	*p*
Intercept	0.81	0.06	0.69; 0.92	**< 0.0001**
MT (baseline)	−0.29	0.05	−0.39; −0.18	**< 0.0001**
Center (worst vs. best)	0.82	0.28	0.26; 1.38	**0.026**
CTG vs. VCMX	0.37	0.12	0.13; 0.61	**0.002**
Timing of soft tissue augmentation (implant uncovering vs. implant placement)	0.03	0.15	−0.26; 0.32	0.515

*Note:* Bold *p*‐values indicate statistically significant.

Abbreviations: CTG, connective tissue graft; MT, peri‐implant mucosal thickness; VCMX, volume stable collagen matrix.

**FIGURE 5:**
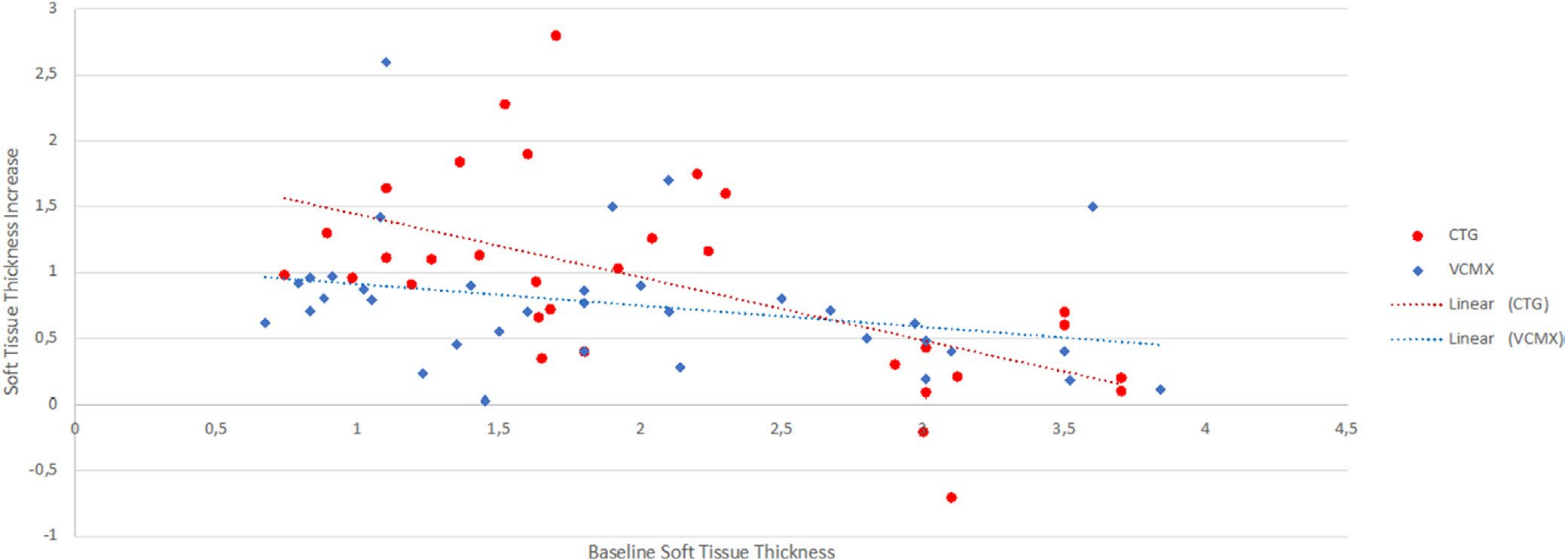
MT changes according to the initial MT. CTG, connective tissue graft; VCMX, volume stable collagen matrix.

Figure 5 shows an explorative model considering the interaction between surgical procedures and baseline MT. For initially thinner soft tissues, CTG displayed better results at the 12 months follow‐up. Nevertheless, in cases of baseline MT ≥ 2 mm, the use of CTG and VCMX yielded comparable results.

CTG showed superior results to VCMX in terms of KMW (0.79 mm, 95% CI: 0.37–1.22, *p* < 0.0001), as shown in Data S1 and S2.

Data S3 shows the results of a sensitivity analysis, stratified in order to evaluate the impact of the timing of the soft tissue augmentation procedure (i.e., implant placement or uncovering). No significant differences were found for MT, KMW, and MBL.

#### 3.2.2 Chair Time and Patient‐Reported Outcome Measures (PROMS)

The mean duration of the surgical procedure was 50.14 ± 13.75 min for CTG and 40.14 ± 11.17 min for VCMX (diff: 10.0 min; 95% CI: 5.02 to 14.98; *p* < 0.0001).

No significant difference was reported for post‐surgical pain (difference: 2.2; 95% CI: −4.3 to 8.7; *p* = 0.51). After 7 days, patients from the CTG group reported an intake of 2.24 ± 2.1 anti‐inflammatory tablets compared with 2.3 ± 2.5 for the VCMX group (difference 0.06; 95% CI: −0.97 to 0.85; *p* = 0.89). Patients allocated to the VCMX group experienced fewer days of discomfort (0.94 ± 1.3 vs. 1.4 ± 1.3; difference 0.46 days, 95% CI: 0.06–0.99, *p* = 0.05). No other significant difference was detected in the post‐operative period.

Patients allocated to both groups showed an excellent final aesthetic and general satisfaction (CTG: 99.28 ± 2.28, VCMX: 98.48 ± 3.92, *p* = 0.22).

## 4 Discussion

Modern clinical research in implant dentistry focused on the critical role of soft tissue at the implant site in order to promote mucosal margin stability and peri‐implant seal and to prevent possible bone resorption (Ericsson et al. 1995) (Berglundh and Lindhe 1996) (Cairo et al. 2019). The addition of soft tissue was also considered very critical at implant sites after GBR, with less risk of final soft tissue recession at the 8‐year follow‐up when soft tissue increase was performed (Cairo, Nieri, et al. 2020).

The present multicenter randomized controlled clinical trial aimed to compare the use of VCMX versus CTG for soft tissue augmentation at implant sites. A total of 98 patients requiring a single dental implant were collected among 4 clinical centers in Italy. At the one‐year follow‐up, the use of CTG was associated with higher performance in terms of MT increase (0.37 mm, 95% CI: 0.13–0.61, *p* = 0.002) and KMW gain (0.79 mm, 95% CI: 0.37–1.22, *p* < 0.0001) compared with VCMX. These outcomes confirmed the high predictability of CTG in improving soft tissue at the implant site, and this procedure should be considered as the gold standard technique (Cairo et al. 2017) (Cairo et al. 2019). Reasons to explain this finding should be related to the very fast process of biological integration of CTG under the flap and to its effectiveness in changing soft tissue shape and contour (Thoma et al. 2016) (Cairo et al. 2017). The magnitude of increase in MT after CTG observed in this study (1.0 ± 0.75) was similar to that reported in other RCTs exploring soft tissue augmentation at the implant site (Thoma et al. 2016) (Cairo et al. 2017) (Hämmerle et al. 2023) (Clem et al. 2024). Furthermore, the final amount of KMW is in accordance with that observed when using CTG (Cairo et al. 2012) (Cairo et al. 2016) or XCM (Jepsen et al. 2013) under coronally advanced flap for root coverage purposes. Moreover, an extensive systematic review on peri‐implant soft tissue corroborated the role of peri‐implant soft tissue, highlighting that soft tissue thickness > 2 mm was associated with less bone loss than thin tissue (Suárez‐López et al. 2016). In the present study, interestingly, both procedures were effective in improving baseline soft tissue conditions, reaching final peri‐implant thickness > 2 mm at 94% of treated sites for CTG and 74% for VCMX, thus suggesting a predictable stability over time.

The present trial confirmed that VCMX was effective in improving peri‐implant soft tissue condition, leading to a significant increase in MT (0.66 ± 0.58 mm) and KMW (0.16 ± 1.1 mm) at one‐year follow‐up. Interestingly, the behavior of matrix and CTG was different during the study, even if a similar flap design was used. While CTG was associated with a progressive increase in MT, leading to an increase of 0.15 mm from 3 to 6 months and a further increase of 0.19 mm at 12 months, VCMX was associated with a progressive reduction in MT, leading to a 0.33 mm loss of thickness between 3 and 6 months and a further 0.13 mm of thickness reduction at 12‐month follow‐up. This performance in this temporal trend was also similar also for KMW changes at different time frames (Figure 4). Reasons to explain this different behavior should be related to the healing process. VCMX was used with its complete thickness (6 mm) during surgery and was susceptible to some shrinkage during the re‐vascularization process, while for CTG we could hypothesize a maturation process leading to soft tissue increase along with its re‐vascularization. Remarkably, VCMX performed in the same manner as CTG when considering baseline MT > 2 mm, thus suggesting that matrix should be considered an excellent treatment option at not very thin baseline tissue, considering its excellent integration and lower morbidity compared with CTG.

Interestingly, no significant difference was reported between the two procedures in terms of final peri‐implant bone levels (MBL for CTG: 0.47 mm; for VCMX: 0.43 mm). Considering the different timings of soft tissue augmentation, different MBL changes could have been expected. However, no differences were found, thus suggesting that soft tissue increase promotes peri‐implant bone stability at one‐year follow‐up (Cairo, Barootchi, et al. 2020) and may prevent MBL (Linkevicius et al. 2010).

Nevertheless, a longer follow‐up is needed in order to evaluate the superiority of one technique over the other (Surdiacourt et al. 2025).

In the present RCT, both procedures were associated with an optimal aesthetic and general satisfaction at the last follow‐up. This observation confirmed that soft tissue surgery determines very frequently an excellent satisfaction in highly motivated patients irrespective of the type of therapy (Cairo, Nieri, et al. 2020). The CTG group reported longer chair time and higher postoperative morbidity in terms of the number of days of discomfort, thus confirming that harvesting procedures at the palatal site may imply a higher challenge for clinicians and patients (Cairo et al. 2012).

In this multicenter RCT, data analysis showed a significant effect center (0.8 mm for primary outcome). This observation confirmed that reconstructive surgery may be associated with a certain level of variability also among expert clinicians and was very similar to what was observed in multicenter studies for periodontal regeneration (Tonetti et al. 2002) (Sanz et al. 2004) where the center effect may account for up to 2 mm in clinical attachment gain. Interestingly, in the present study, the effect center was similar for both groups, thus suggesting that the magnitude of the effect was much more related to flap and wound management than CTG or VCMX per se. This element should be considered when assessing the generalizability of these outcomes.

### 4.1 Limits of the Study

This study failed to find worse clinical results in smokers. Very few patients were heavy smokers (4.1% CTG and 6.1% VCMX group), and great attention was given suggesting the patients avoid smoking during the early healing phases. This might have reduced the possibility of clearly analyzing the impact of smoking, especially for the VCMX.

The grafts were used both during implant placement and implant uncovering, and there was a very slight, non‐significant difference of 0.03 mm favoring the graft placed at implant uncovering. Considering that this difference might not be clinically relevant, these data suggest that both techniques are effective in gaining MT around dental implants.

Another limitation of the study is the variability in implant brands, abutment dimensions, and locations of the procedure. Despite no differences between groups being found at baseline, this might have had an impact on MT gain. Similarly, the prosthetic restorations were carried out by means of different materials and designs, according to the preference of the clinicians: depending on the location where pressure is applied, the soft tissue thickness at a set level may have been influenced by the prosthetic procedure. Further studies are needed to address the impact of prosthetic conditioning on the final MT gain.

In conclusion, this multicenter RCT suggested:

- Both VCMX and CTG improved soft tissue conditions at implant site
- CTG yielded better outcomes in terms of MT and KMW
- VCMX was associated with shorter chair‐time and less post operative discomfort, but both procedures achieved excellent final patient satisfaction

## Author Contributions

**Francesco Cairo:** conceptualization, investigation, funding acquisition, writing – original draft, methodology, validation, visualization, writing – review and editing, data curation, supervision. **Cosimo Rupe:** investigation, writing – original draft, formal analysis, methodology. **Raffaele Cavalcanti:** investigation, validation, visualization. **Luca Landi:** investigation, validation, visualization. **Antonio Rupe:** investigation, validation, visualization. **Nicola Marco Sforza:** validation, visualization. **Walter Castelluzzo:** formal analysis, writing – review and editing, data curation. **Maria Di Martino:** resources, data curation, writing – review and editing. **Luigi Barbato:** data curation, resources, conceptualization, writing – original draft, writing – review and editing, validation, visualization.

## Conflicts of Interest

The authors declare no conflicts of interest.

## Supporting information


**Data S1.** clr70050‐sup‐0001‐AppendixS1.docx

## Data Availability

The data that support the findings of this study are available on request from the corresponding author. The data are not publicly available due to privacy or ethical restrictions.
