# Supplementary material for: Cross‐Linked Volume‐Stable Collagen Matrix Versus Connective Tissue Graft for Soft Tissue Augmentation at Implant Site. A Non‐Inferiority, Multicenter Randomized Clinical Trial
Source: Clin Oral Implants Res. 2025 Sep 22;37(1):45–56. doi: 10.1111/clr.70050 (PMC12767552; doi:10.1111/clr.70050)
Supplement: Supplementary file 1 — Data S1. clr70050‐sup‐0001‐AppendixS1.docx [file CLR-37-45-s001.docx]

| Variables | Estimate | Std Error | 95% CI | P value |
| --- | --- | --- | --- | --- |
|  |  |  |  |  |
| Intercept | 0.52 | 0.11 | 0.31; 0.73 | **<0.0001** |
| KMW (Baseline) | -0.27 | 0.09 | -0.47; -0.07 | **0.007** |
| Center (Worst vs Best) | 1.26 | 0.34 | 0.59; 1.93 | **0.005** |
| CTG vs VCMX | 0.79 | 0.21 | 0.37; 1.22 | **<0.0001** |
| Timing of Soft tissue augmentation  (Implant uncovering vs Implant Placement) | 0.17 | 0.27 | -0.36; 0.7 | 0.696 |

**Supplementary Materials 1**. Results of the Multivariate analysis for change in KMW between baseline and 12 months follow-up. **KMW**: Keratinized Mucosa Width; **CTG:** Connective Tissue Graft; **VCMX**: Volume Stable Collagen Matrix.

**
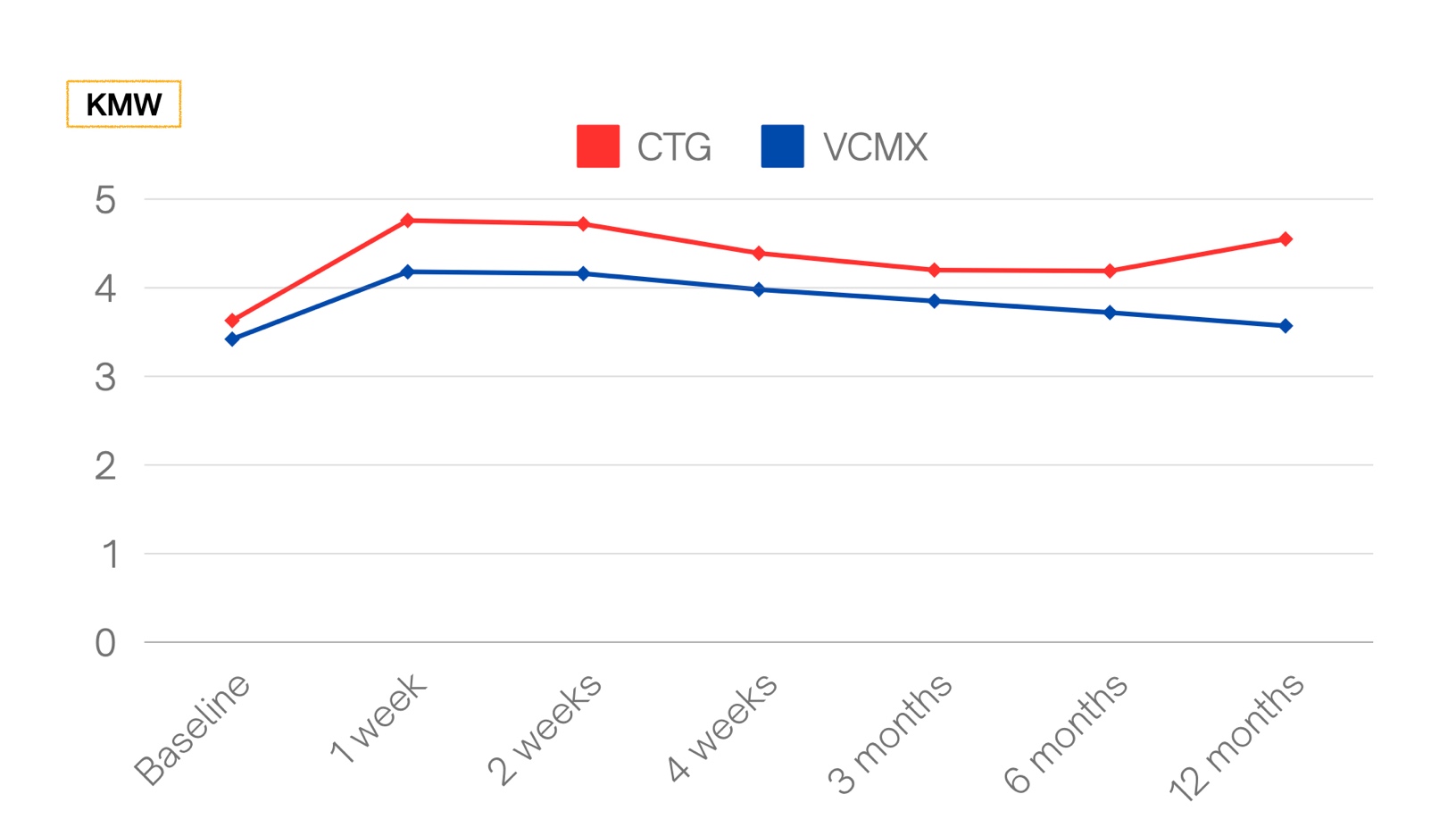
**

**Supplementary Materials 2.** KMW changes at each interval (**CTG**, connective tissue graft; **VCMX**: Volume Stable Collagen Matrix; **KMW**: Keratinized Mucosa Width).

| Treatment | Timing of Soft Tissue Augmentation procedure | MT Augmentation  (Mean, SD) | p-value | KMW Augmentation  (Mean, SD) | p-value | MBL  (Mean, SD) | p-value |
| --- | --- | --- | --- | --- | --- | --- | --- |
|  |  |  |  |  |  |  |  |
| CTG | Implant Placement | 1.11 (0.17) |  | 0.58 (1.11) |  | 0.71 (0.67) |  |
|  | Implant Uncovering | 0.94 (0.63) | 0.44 | 1.06 (1.12) | 0.17 | 0.34 (0.85) | 0.12 |
|  |  |  |  |  |  |  |  |
| VCMX | Implant Placement | 0.75 (0.73) |  | 0.02 (1.31) |  | 0.5 (1) |  |
|  | Implant Uncovering | 0.59 (0.45) | 0.34 | 0.30 (0.96) | 0.32 | 0.38 (0.97) | 0.66 |
|  |  |  |  |  |  |  |  |

**Supplementary Materials 3.** Changes in KMW, MT, MBL stratified for timing of soft tissue augmentation procedure (**CTG**, connective tissue graft; **VCMX**: Volume Stable Collagen Matrix, **MT**: peri-implant mucosal thickness, **KMW**: Keratinized Mucosa Width, **MBL**: Marginal Bone Loss). ANOVA Test was used to test the differences between groups.
